# Global Methylation in Exposure Biology and Translational Medical Science

**DOI:** 10.1289/ehp.1103423

**Published:** 2011-06-13

**Authors:** Heather H. Nelson, Carmen J. Marsit, Karl T. Kelsey

**Affiliations:** 1 Masonic Cancer Center, and; 2 Division of Epidemiology and Community Health, University of Minnesota, Minneapolis, Minnesota, USA; 3 Department of Pathology,; 4 Center for Environmental Health and Technology, and; 5 Department of Epidemiology, Brown University, Providence, Rhode Island, USA

**Keywords:** ALU, biomarker, epigenetic, exposure biology, LINE-1, methylation

## Abstract

Background: Many groups are actively investigating how the epigenetic state relates to environmental exposures and development of disease, including cancer. There are myriad choices for capturing and measuring the epigenetic state of a tissue, ranging from assessing the total methyl-CpG content to array-based platforms that simultaneously probe hundreds of thousands of CpG loci. There is an emerging literature that uses CpG methylation at repetitive sequences, including *LINE-1* (long interspersed nuclear element-1) elements, to capture the epigenomic state.

Objectives: We explored the complexity of using CpG methylation at repetitive sequences in epidemiology and translational medical research and suggest needed avenues of research to clarify its meaning and utility.

Conclusions: Among the most urgent avenues of research is the need for prospective studies to eliminate the possibilities of reverse causality, and development of new *LINE-1* assays that capture both class of *LINE-1* element and copy number.

Epigenetics describes mechanisms of mitotically heritable changes in gene expression that occur via means other than DNA sequence alteration. The proper establishment of the individual cellular epigenetic code is critical for proper development. Further, it has been hypothesized that improper establishment of the epigenetic code can translate to disease development, including loss-of-imprinting disorders and cancer (where this scientific field is most significantly developed) and, more recently, disease-associated states occurring as the result of epigenetic alterations of the germline, termed “epimutations” (Bennett et al. 2010; Dobrovic and Kristensen 2009; Hitchins 2010; Wong et al. 2010). The establishment of the epigenetic state that will guide development begins immediately after fertilization when the nascent organism loses all DNA methylation marks and resets the entirety of its epigenetic condition in a cell-type–specific fashion in order to dictate somatic development. Hence, the fetal environment is generally accepted to be crucially important in the genesis of proper epigenetic marks and in the subsequent orchestration of complex cellular and organismal differentiation. Further, it is likely that the intrauterine environment’s hypothesized role in affecting health and disease later in life results from perturbation of epigenetic developmental programming (Tamashiro and Moran 2010).

The repertoire of epigenetic mechanisms is complex and not yet completely understood. However, histone proteins, which interact with DNA to form chromatin, are central participants in epigenetic regulation. Posttranslational modification of histone proteins is thought to alter the structure of chromatin, allowing various processes to occur that have profound consequences for the utility, parsimony, and general long-term viability of the genome, including transcription, DNA repair, DNA replication, and gene silencing. The expression or repression of the genome is marked by appropriately modified histones, and this is reflected at the DNA level as the absence or presence of CpG dinucleotide methylation. CpG dinucleotides are significantly underrepresented in the genome (likely due to the spontaneous deamination of cytosine), but when present they are often concentrated in CpG islands. CpG islands are associated with almost 50% of all described genes, and most are located in gene promoter regions. DNA methylation, as a representative of epigenetic regulation, is significantly more amenable to measurement in epidemiologic studies than are histone modifications, and this is reflected in the emerging literature. The simplicity of measuring DNA methylation is attributable to the use of sodium bisulfite conversion, a chemical process that deaminates unmethylated cytosines to uracil while retaining methylated cytosines (Herman et al. 1996). DNA methylation determination is then made by distinguishing between a C or T residue, and any genotyping platform may be used to assess methylation after bisulfite conversion of the DNA. With the advent of more affordable high-throughput sequencing technologies, other methods not relying on sodium bisulfite, such as methylated DNA immunoprecipitation, are beginning to be used in population-based contexts and will likely grow in utility.

The importance of maintaining the epigenetic state of individual genes is most clearly illustrated for the occurrence of cancer. A solid body of research describes a vast array of tumor suppressor genes inactivated by a process that includes gene promoter hypermethylation in almost every type of cancer. However, even before we understood the role of tumor suppressor promoter hypermethylation, researchers recognized that solid tumors are heavily hypomethylated relative to their normal tissue counterparts. This genomic or global hypomethylation is now believed to occur early in tumorigenesis, even in precancerous lesions (Pufulete et al. 2003; Suter et al. 2004), potentially accelerating the genomic instability currently thought to be necessary for cancer development. Yet “global methylation” is a vague term, used to interchangeably refer to various measures of cytosine methylation of the genome overall, of repetitive elements, or of multiple gene regions. In fact, these are potentially noncomparable measures arising from distinct cellular processes; therefore, greater clarity is called for when examining and reporting these disparate measures.

## Measuring Global Methylation

Because there are critical links between genomic hypomethylation and pathogenesis, there is a growing research interest in determining whether changes to the global status of DNA methylation is related to the environment and whether these changes can be biomarkers of disease. A number of methods have been developed to quantify global methylcytosine content of DNA samples. Total methylcytosine can be directly measured using chromatographic methods after digestion of the DNA into single nucleotides. Although these methods are highly quantitative, they generally require large amounts of DNA and highly specialized equipment and are generally difficult to standardize for use on large sample collections such as those collected in an epidemiologic context. Thus, many research groups have opted for polymerase chain reaction (PCR)-based methods that capitalize on bisulfite modification chemistry. Weisenberger et al. (2005) described methylation at satellite repeats, *LINE-1* (long interspersed nuclear element-1), and *ALU* elements as being reasonably well correlated with total methylcytosine content using a quantitative PCR-based approach. This effort followed a report by Yang et al. (2004) that described the initial use of bisulfite sequencing of *LINE-1* and *ALU* elements to determine global methylation. These studies prompted a new line of investigation using quantitative methylation at repetitive elements in lymphocytes to evaluate the association of variation in environmental exposures with levels of what became accepted as a measure of global DNA methylation. For example, many groups have evaluated global methylation and air pollution, with most of the studies observing decreased methylation among exposed individuals (Baccarelli et al. 2009; Bollati et al. 2007; Tarantini et al. 2009), with the exception of one investigation of coke oven workers who had elevated global methylation relative to controls (Pavanello et al. 2009). It should be noted that in a recent study, Choi JY et al. (2009) were unable to replicate the correlation between total methylcytosine content and *LINE-1* methylation measured with bisulfite pyrosequencing.

*Methylation at* LINE-1 *and* ALU. If the methylation status of LINE-1 and ALU elements is to be used to signify global methylcytosine content, it is important to understand what these elements represent and why they are methylated in nondiseased tissues. Nearly half the DNA content of the human genome consists of repetitive sequences of DNA: transposons, retrotransposons, and endogenous retroviruses. Typically these elements are nontranscribed and maintained as heterochromatin (and hence characterized by hypermethylation). Most of these elements are devoid of phenotype through truncation or other inactivating mutations. Activation of transposons, however, can initiate or enhance disease processes, including carcinogenesis. In fact, transposons have been exploited as tools for mammalian mutagenesis and forward genetics screens (Largaespada 2009; Ostertag and Kazazian 2001; Starr et al. 2009). *LINE-1* elements are retrotransposon sequences, whereas *ALU* elements are short interspersed nuclear elements. Together, the *LINE* and *ALU* sequences comprise approximately 30% of the genome (Miki 1998). Their ubiquitous presence genomically, combined with their relatively conserved sequence and propensity of methyl-CpG targets, highlights their appeal as a representative measure of global methylation.

However, further examination of these sequences reveals significant technical limitations that must be considered carefully. There are approximately 500,000 *LINE-1* elements in the genome, and it is unknown how many of these are of full length (6 kb), consisting of *a*) a 5´ untranslated region (UTR) with an internal RNA polymerase II promoter, *b*) two open reading frames encoding an RNA binding protein and elements necessary for retrotransposon activity, and *c*) a 3´ UTR containing a polyadenylation signal (Cordaux and Batzer 2009). The CpG sequence targeted by pyrosequencing for *LINE-1* is in the 5´ region (usually three to six sites); however, the 5´ end of the sequence tends to be deleted (but with unknown frequency) except in the more active, evolutionarily newer sequences. Therefore, with this assay we cannot, with accuracy, know how many elements we are evaluating or whether this number is similar across samples or individuals. Simply put, the denominator in this measurement is not known and is likely not constant. For example, sex is associated with *LINE-1* methylation (Wilhelm et al. 2010; Zhu et al. 2010). One cannot know (with certainty) whether this sex difference is attributable to copy number variation in *LINE-1* on the X and Y chromosomes or a sex hormone effect that affects methylation. Further complicating this measurement problem is the knowledge that a subset of *LINE-1* elements (possibly up to 80) is transposon competent, meaning that when these competent sequences are unmethylated (i.e., immediately after fertilization) they may generate new *LINE-1* sequences by reintegrating into the genome. In fact, evidence is building that humans are highly polymorphic for *LINE-1* sequences (Ewing and Kazazian 2010); therefore, it is almost certain that populations are evolving differently with regard to the distribution of *LINE-1* sequences.

Assessment of *ALU* methylation is subject to many of the same concerns as *LINE-1*. Unlike *LINE-1*, *ALU* uses an internal RNA polymerase III promoter and lacks any coding sequence. Instead, for retrotransposition, *ALU* elements require the use of the retrotransposition machinery of *LINE-1* (Dewannieux et al. 2003). Therefore, the measured CpGs at *ALU* are likely under different selective pressures relative to the inactive and active *LINE-1* sites. In fact, the quantitative assessment of DNA methylation at *ALU* is consistently about one-third to one-fourth the level of methylation at *LINE-1*. This supports the notion that methylation at *LINE-1* and *ALU* might measure something quite different. Similar to *LINE-1*, *ALU* is highly polymorphic, again leading to concerns regarding population differences in the number and location of the repeats. Finally, DNA methylation occurs throughout the genome in a sequence-context–dependent fashion, and the extent to which regional sequence context might affect different measures of DNA methylation remains to be assessed.

Finally, the degree of methylation at repeat regions reflects the “stability” of an individual’s genome because repeat sequences are recognized, in many cases, to harbor known fragile regions and disease-associated expression (Belancio et al. 2009; Dion et al. 2008; Kulis and Esteller 2010). There is little direct evidence that the known hypomethylation that occurs in tumor cells is responsible for the commonly observed genomic instability that can similarly be a hallmark of the malignant phenotype, although this concept is largely becoming accepted as an important thesis.

Despite these limitations, there are consistent epidemiologic observations in the literature that support methylation at *LINE-1* and *ALU* as meaningful biomarkers. The limited number of published assessments of these two distinct measures in the same individual in the same tissue suggest that the measures are weakly but significantly correlated (Chalitchagorn et al. 2004; Choi SH et al. 2009; Weisenberger et al. 2005), although few data describe variation across tissues or from the same individual at various time points.

*Evidence that DNA methylation at* LINE-1 *and* ALU *is a biomarker of environmental exposures and disease states.* Early epidemiologic studies assessing DNA methylation at *LINE-1* and *ALU* assumed that these measures should both reflect the state of global methylation. In fact, the assumption that the level of methylation at DNA repeat sequences reflected some individually relevant set point with “global” implications was largely untested. However, the average methylation at these repetitive elements in blood cells is correlated with aging (Bollati et al. 2009; Jintaridth and Mutirangura 2010; Kim KY et al. 2010; Zhu et al. 2010); race/ethnicity (Terry et al. 2008); and many environmental exposures, including air pollution (Baccarelli et al. 2009; Bollati et al. 2007; Tarantini et al. 2009), metal exposure (Pilsner et al. 2010; Wilhelm et al. 2010; Wright et al. 2010), persistent organopollutants (Kim KY et al. 2010; Rusiecki et al. 2008), and alcohol consumption (Choi JY et al. 2009; Zhu et al. 2010). Global methylation is also associated with disease states, including many cancers (Cho et al. 2010; Choi JY et al. 2009; Hou et al. 2010; Moore et al. 2008; Ting Hsiung et al. 2007; Wilhelm et al. 2010), stroke (Baccarelli et al. 2010; Kim M et al. 2010), and heart disease (Baccarelli et al. 2010; Kim M et al. 2010; Smolarek et al. 2010). Interestingly, there seems to be little coherence in the relationship of *LINE-1* methylation with methylation at *ALU* repeats in revealing associations of exposures or disease states. This fact has received little attention and remains essentially unexplained. For example, there is a consistent relationship between persistent organic pollutant exposure and hypomethylation at *ALU* but not at *LINE-1* (Kim KY et al. 2010; Rusiecki et al. 2008), and the reason for this dichotomy is not understood.

Inconsistencies in the literature on the global methylation–disease relationship may be attributable to measurement method. For example, in two well-regarded studies, risk of stroke is associated with either global hypomethylation, as assessed by *LINE-1* bisulfite pyrosequencing (Baccarelli et al. 2010), or global hypermethylation, as assessed using MethyLight at *ALU* and *SAT2* (spermidine/spermine *N*^1^-acetyltransferase family member 2) (Kim M et al. 2010). However, for other diseases (e.g., bladder cancer) the relationship between global methylation and disease is clear. In initial work in the Spanish Bladder Cancer Study, Moore et al. (2008) assessed blood cell methylation using a combination of high-performance capillary electrophoresis and methylation-sensitive restriction enzyme digestion and densitometry. These authors reported very elevated bladder cancer risks in nonsmokers with low levels of DNA methylation. In subsequent work in the New Hampshire bladder cancer study, Wilhelm et al. (2010) reported consistent results using pyrosequencing of *LINE-1* from blood-derived DNA. In more recent work in the Shanghai Bladder Cancer Study, again measuring DNA methylation at *LINE-1* using pyrosequencing, Cash et al. (2011) reported additional evidence of the bladder cancer risk associated with lower levels of DNA methylation (primarily in nonsmokers).

The first prospective studies of the association of repetitive element DNA methylation and disease have been reported only recently. Kim M et al. (2010) demonstrated that increased methylation, measured using *ALU* and *SAT* repeat regions, was associated with cardiovascular disease risk. Baccarelli et al. (2010) found that *LINE-1* DNA methylation level (assessed using pyrosequencing) prospectively predicts ischemic heart disease. In addition, there is a significant correlation between maternal and newborn *LINE-1* levels, consistent with other work suggesting that heritability of *LINE-1* methylation level may be sex specific (Kile et al. 2010; Mirabello et al. 2010). This highlights (although clearly does not in any way prove) the possibility that some component of *LINE-1* DNA methylation level may be genetically determined.

*Utility of animal models.* Important work in animals addresses some aspects of these questions. Work in the agouti mouse model has confirmed that environmental alterations during pregnancy (most prominently in diet) affect epigenetically determined phenotypes in offspring, reportedly through the altered methylation status of intracisternal A-particle (IAP) retrotransposons upstream of the agouti locus (Waterland and Jirtle 2003; Wolff et al. 1998). *In vitro* and *in vivo* animal studies also have shown that global methylation is a consequence of arsenic exposure (Davis et al. 2000; Zhao et al. 1997), although this is generally measured using methods that are not analogous to assessing repeat methylation in humans and at exposures that are not relevant to human exposures. Although these animal models do not prove that global methylation effects lead to disease outcomes caused by arsenic, it may remove questions of reverse causality and provide insight on the mechanism of arsenic’s toxicity.

However, humans and rodents have important systematic differences regarding repeat regions. Interspersed repetitive elements make up some 45% of the human genome (Lander et al. 2001) compared with approximately 38% of the rodent genome (Waterston et al. 2002). Retrotransposons can be divided into two distinct groups: those containing long-terminal repeats (LTRs) and others (so-called non-LTR retroelements). LTR retrotransposons have been inactive in humans for millions of years but make up approximately 8% of the human genome (Lander et al. 2001). This contrasts with rodent genomes, in which the LTR elements are active and are known to be responsible for significant numbers of germline-associated mutations (reviewed by Maksakova et al. 2006). Actively transposing repeats are believed to be responsible for about 10% of mutations in rodents (Maksakova et al. 2006), unlike in humans, where this number is considerably smaller (Belancio et al. 2009). The activity of transposable elements has often been assumed to be confined to the developing embryo and possibly to cancer cells in humans (Branciforte and Martin 1994; Ergun et al. 2004; Martin 1991; Martin and Branciforte 1993). However, LINE-1 proteins (ORF1 and ORF2) can be found in human somatic tissues (Ergun et al. 2004). Further, somatic LINE-1 retrotransposition also occurs in transgenic mouse models (Babushok et al. 2006; Kano et al. 2009), and transgenic mice have significantly greater LINE-1 mobilization in somatic tissues than in the germline (An et al. 2008; Babushok et al. 2006). Thus, rodents and humans both display repeat region hypermethylation but have evolutionarily very different systems mediating these epigenetic changes. Hence, care is needed when generalizing observations in rodents to humans.

*Surrogate tissues.* For epidemiologists, there is an obvious question posed by the literature: What are we measuring with these different assays? As important as this question is, assessing global methylation raises yet another, more classic problem: Is global DNA methylation (measured in each of these seemingly distinct assays) different in different tissues? Of course, it is unclear how to actually study methylation levels in cells from inaccessible tissue. Often we turn to surrogate tissues, such as circulating blood lymphocytes or sloughed buccal cells. Using these surrogate tissues to evaluate gene-specific methylation is complicated by the fact that epigenetic marks can occur in a tissue-specific manner (Christensen et al. 2009).

Although tissue-associated differences in DNA repeat methylation have been described in some studies, exhaustive research remains to be performed. The current literature supports the assertion that there are tissue differences in *LINE-1* and *ALU* DNA methylation levels, but these are not large, and they may be influenced by the environment (Chalitchagorn et al. 2004; Choi SH et al. 2009; Wu et al. 2011a, 2011b; Zhu et al. 2010). The mechanism(s) responsible for these tissue-specific differences is unknown. Recent work shows that *LINE-1* retrotransposition can occur in a tissue-specific fashion (Muotri et al. 2010), although whether this would be sufficient to affect the measure of *LINE-1* DNA methylation overall is not known. Because it is clear that different tissue development is attributable to differential gene expression, born of distinct epigenetic profiles (Christensen et al. 2009), differences in repeat region methylation might be the result of set point differences that are determined *in utero* and are tissue specific. Alternatively, they could be differentially induced over time by changes in the local environment of each tissue, or they might be affected by differences (environmental or genetic) in maintenance DNA methyltransferase or de novo DNA methyltransferase. It should be noted that DNA methylation at the fifth carbon position of cytosine is covalently bound, mitigating concerns that this has limited stability. In sum, detailed experiments should be conducted in order to further understand the contribution of each of these possibilities to the measureable levels of repeat region DNA methylation, because this will enhance the interpretability of these measures.

There is evidence that the spectrum of cell types present in peripheral blood affects measures of *LINE-1* and *ALU* DNA methylation (Zhu et al. 2010). This is consistent with the notion that tissues are different with respect to *LINE-1* methylation in that the white blood cell lineages are distinct and their maturation and differentiation are driven by epigenetic means (similar to other tissues). This clearly implies that studies using peripheral blood as the tissue of interest for measuring global methylation must take into account the dynamic nature of the profile of the tissue and any somatic condition known to alter this profile. Of course, because white blood cells infiltrate many tissues, cellular heterogeneity is almost certain to be a feature of any tissue measure that is used in epidemiologic study. Variation or even bias associated with these measures must be considered in study design and analysis.

Additional work is needed to describe the quantitative variation in the different measures of global methylation. The factors that affect variation in global methylation are not completely understood, and the kinetics, relative magnitude, and precise relationship of different measures of DNA methylation among tissues remain to be well defined. More investigation will help us to understand how well alterations of DNA methylation at repeat regions (and other global methylation sites) in an accessible tissue (e.g., blood) reflect processes that are occurring in other tissues or represent systemic changes allowing for inference on disease risk or prevention. Clearly, the known disease associations of altered imprinting and epimutation suggest that some disease processes may manifest in all cells and hence be quite detectable in blood.

*Establishment of methylation marks.* Inherent in the understanding that there is reproducible tissue specificity in measures of methylation is the concept that methylation marks, then, must have been initially differentially established during tissue differentiation and subsequent development. The “fetal origins” or “developmental origins and health and disease” hypothesis was developed from a series of studies that demonstrated an association between measures of birth size and long-term chronic disease risk. Most of these studies focused on cardiovascular disease and metabolic syndromes (Barker and Bagby 2005; Barker and Osmond 1988; Barker et al. 1989), linking antenatal environmental factors, including diet, xenobiotic exposures, stress, and lifestyle factors, to altered fetal growth and—through programming—to permanent biological and physiologic changes in the offspring. The mechanism of this reliably observed phenomenon is necessarily epigenetic in nature. This link between antenatal exposures and altered phenotypes supports the hypothesis that observed variable set points of global methylation in specific tissues is related to the *in utero* environment. Further, it is likely that these differences can be detected at birth and that the environment encountered postnatally affects variability, although research is only beginning to examine this variability and the effects of various periods of life on epigenetic regulation.

The variability in the levels of methylation globally or at specific repetitive elements defined at birth may, in fact, explain the regional differences in overall levels of methylation of *LINE-1* that have been observed in various studies but have received little discussion. For example, Zhu et al. (2010) examined *LINE-1* methylation related to exposures in five studies from Europe and the United States; the largest observed effect was study site, despite the fact that a single laboratory conducted *LINE-1* methylation measurements. Similarly, comparing data from two bladder cancer studies, one in New Hampshire (Wilhelm et al. 2010), and one in Shanghai, China (Cash et al. 2011), again collected from a single laboratory, Wilhelm et al. (2010) demonstrated significant differences in the levels of *LINE-1* methylation between populations. The underlying etiology of these differences is not clear, although it could be related to genetic differences in the populations or differences in *in utero* or postnatal environments encountered by these individuals. Although such differences can be accounted for in multivariable models, understanding the nature of these differences may be critically important to interpreting the biological meaning of exposure- or disease-associated changes in *LINE-1* or other repetitive element measures of global methylation.

*Other repetitive sequences. LINE-1* and *ALU* are not the only repetitive elements that have been used as determinants of global methylation. DNA sequence repeat regions are found across the genome and include satellite repeated sequences found in tandem that have arisen as a result of amplification of simple repeats (these include *SAT*α in centromeric regions, and *SAT2* and *SAT3*, which are subtelomeric) (Jordan et al. 2003; Lander et al. 2001). The DNA transposons are the oldest type of transposable element and are largely completely degenerate as a result of deletion and truncation events over time. However, the *SAT* elements are small (averaging 215 bp), a few remain active in the genome, and their methylation has been poorly studied in carcinogenesis (Alexander et al. 2010). Although *LINE-1* and *ALU* are considered retrotransposons, others of the LTR type, considered endogenous retroviruses, have been used to examine genomic methylation state. More than 400,000 of these retroviral elements encompass 8% of the human genome (Lander et al. 2001). The inappropriate activation of these elements has been linked to various cancers, particularly Hodgkin’s lymphoma, as well as in autoimmune diseases such as systemic lupus erythematosus, although their causality for disease is not yet established (Balada et al. 2009; Florl et al. 1999; Januchowski et al. 2004; Menendez et al. 2004; Ogasawara et al. 2003; Okada et al. 2002; Ruprecht et al. 2008; Stacey and Sagulenko 2010; Stengel et al. 2010). The LTR elements have been subject to limited study, but their potential for activation associated with hypomethylation and the determinants of that hypomethylation remain largely unexplored (Gimenez et al. 2009; Huh et al. 2008; Schulz et al. 2006).

## Conclusions and Research Needs

Current research strongly suggests that assessment of the level of DNA methylation at repeat regions and in the genome as a whole is poised to reveal crucially important biological processes that are causal or contributory in numerous disease states. There are several caveats, however, to moving this line of research forward, specifically regarding the molecular phenotype of these alterations, their link to disease processes, and methodologies capable of distinguishing differences in methylation extent from inherent genomic variability of these elements.

Environmentally induced alterations in DNA methylation at repeat sequences may have specific and definable phenotypic consequences. One can speculate that either stochastic or targeted hypomethylation at repeats could activate small noncoding RNAs that are normally silent. This activation could then induce or enhance toxicant action. Indeed, quantifying or measuring this kind of biological effect might easily differ depending upon the method used to assess hypomethylation (e.g., some methods interrogate the important small RNA sequences, whereas others do not), consistent with the current literature. Alternatively, global hypomethylation may act chiefly to destabilize the genome. Instability might contribute to numerous important disease phenotypes, particularly if it is targeted. In either of these constructs, hypomethylation at repeat regions would have very different phenotypic consequences than epigenetic alteration of specific genes but could potentially represent an important part of the toxicant mode of action. In our effort to better understand global hypomethylation, we must first define the biological mechanisms that control and define this potentially very important phenomenon. At present, we would argue that there is really no way to generate a reasonable consensus on the meaning of “global DNA methylation.” Although this concept is attractive and potentially useful, it really has no transcendent meaning. As we move toward intensive research of this concept, it seems prudent to use more specificity in describing the manner in which we assess DNA methylation.

In addition, there are few prospective studies and a clear need for biological experimental follow-up to actually determine the mechanism and import of the observations that the environment alters the epigenetic state. Examinations determining how measures of these alterations in surrogate tissues relate to changes in the disease-targeted tissues will provide a clearer understanding of whether it is the changes in target tissues or potentially in the surrogate tissues themselves that are playing a contributory role in disease. Until we have an appropriate grasp on the underlying meaning of the variability of these measures and the factors effecting their measurement, we cannot make conclusive or useful recommendations to affect these measures for the sake of disease prevention or treatment efforts.

Assessing *LINE-1* methylation with the current PCR-based protocols is perfected to the point that very large studies are now possible; this is an exciting and important development. However, the assays have some unique features. The *LINE-1* assays yield a measure that is restricted to *LINE-1* regions that have an intact internal promoter. Recent research is probing human and model systems to define the likely significant contribution of active retrotransposition (occurring during blastocyst development) to disease (Dean et al. 2005). Active elements may have a biology that is quite unique, and the selective assessment of these regions may result in the study of a distinct group of phenotypically unique regions. That is, inactive *LINE-1* repeats that are not transposition competent or are truncated may behave in a systematically different manner from the active sequences. This bias in our assessment of DNA methylation may be critically important and drive some of the associations observed in epidemiologic inquiry. Some form of phenotypic selection also might explain the differences observed in studying *ALU* and *LINE-1* regions in the epidemiologic context (and potentially even help to explain within-person variability in these measures). Hence, designing assays that can differentially assess DNA methylation in active and nonactive *LINE-1* regions would be revealing. Clearly, we are in urgent need of a better understanding of the relationship of *LINE-1* methylation levels with active retrotransposition-competent compared with retrotransposition-incompetent regions, with the idea that this may result in some important form of phenotypic selection.

As noted above, there is good evidence that *LINE-1* regions are polymorphic in humans. The impact of this polymorphism on health and disease is essentially unstudied. As we continue to assess the epigenetics of the *LINE-1* region, it is important that we devise methods to assess the extent and impact of this variation. Indeed, more assessment of the epidemiologic importance and potential variation at the other DNA repeat regions would also be of interest. Limited study of tandem repeats and LTR transposons has been reported, but there is currently little systematic comparison of these measures in humans. Because genomewide CpG-specific array-based assays are now more commonly used in population-based studies, there is also a need to determine whether data from these arrays can be used to assess genomewide methylation. Understanding the coverage of the arrays and selection of sites for examination is critical, because these measures may reflect underlying selective pressure for some level of methylation dependent on the sites measured. Clearly, multidisciplinary approaches spanning epidemiology, genetics, biostatistics, and bioinformatics are needed to best study methylation as a marker of exposure and disease and to interpret data from these examinations.

In summary, we believe the term “global methylation” should be reserved for non-sequence-dependent determinations (specifically analytical measures) of methylcytosine content. Other quantitative assessments of DNA methylation dependent on sequence content should be described specifically according to this attribute and interpreted relative to possible phenotypic consequences.
